# A retrospective study on the influence of siblings’ relatedness in Bolivian patients with chronic Chagas disease

**DOI:** 10.1186/s13071-019-3518-4

**Published:** 2019-05-24

**Authors:** Juan Espinosa-Pereiro, Adrián Sánchez-Montalvá, Fernando Salvador, Augusto Sao-Avilés, Elena Sulleiro, Israel Molina

**Affiliations:** 1 Infectious Diseases Department, Vall d’Hebron University Hospital, Programa de Salut Internacional de l’Institut Català de la Salut (PROSICS), Barcelona, Spain; 2 Vall d’Hebron Resarch Institute, University Hospital Vall d’Hebron, Barcelona, Spain; 3 Cardiac Imaging Unit, University Hospital Vall d’Hebron, Barcelona, Spain; 4 Microbiology Department, University Hospital Vall d’Hebron, Universitat Autònoma de Barcelona, Barcelona, Spain

**Keywords:** Chagas disease, Siblings, Brotherhood, Cardiomyopathy, Gastrointestinal disease, Delayed type hypersensitivity

## Abstract

**Background:**

Chagas disease is a protozoan infection caused by *Trypanosoma cruzi*. The disease has a chronic course in which 20–30% of the patients would develop progressive damage to the cardiovascular system and the gastrointestinal tube. We are still unable to predict who will develop end-organ damage but there are some acquired and genetic risk factors already known.

**Results:**

We reviewed data from 833 patients with serologically confirmed Chagas disease in this retrospective study. Patients were classified as siblings or non-siblings (controls) and the results of pre-treatment blood PCR assay, end-organ damage (cardiac and/or gastrointestinal), and the presence of delayed type hypersensitivity (DTH) skin involvement in patients treated with benznidazole were analyzed. Siblings were grouped by family and we randomly generated groups of 2 or 3 persons with the remaining controls. We classified the results of each variable as concordant or discordant and compared the concordance in these results among the sibling groups with that among control groups. We identified 71 groups of siblings and randomly generated 299 groups of non-related patients. Pre-treatment blood PCR concordance was significantly higher (19%) among siblings compared to controls (*P* = 0.02), probably due to a higher frequency in pre-treatment positive results. No other statistically significant differences were found.

**Conclusions:**

A significant difference was found in the concordance of pre-treatment blood PCR for *T. cruzi* among siblings compared to non-related controls.

**Electronic supplementary material:**

The online version of this article (10.1186/s13071-019-3518-4) contains supplementary material, which is available to authorized users.

## Background

Chagas disease is caused by the protozoan hemoflagellate *T. cruzi* and is estimated to affect more than 6–13 million people worldwide. Socioeconomic development and migrant flows have caused the disease to change from a rural vector-borne disease endemic to Latin America to a global disease with different mechanisms of transmission (mother-to-child transmission, tissue and organ transplantation, blood transfusion, and oral transmission through infected food). After the initial, acute phase, Chagas disease has a chronic course in which most patients remain asymptomatic for life, the so-called indeterminate phase of the disease. Over years to decades, about 20–30% of these patients progress to the determinate phase of the disease, characterized by cardiac and/or gastrointestinal manifestations [1, 2].

In chronic Chagas disease, there is a consensus about the recommendation of treating patients younger than 18 years-old, women at childbearing age, and people at risk or actually suffering a reactivation of the disease (i.e. when receiving immunosuppressant drugs), on the basis of different studies [3–5]. The BENEFIT study has recently showed no improvement when there is established chronic chagasic cardiomyopathy [6]. The cornerstone of the treatment against *T. cruzi* is benznidazole. The main drawback of benznidazole is its toxicity, which appears in 40–70% of the patients leading to discontinuation of treatment in 12–18% of them [7–9].

There is increasing evidence on predisposing factors to organ damage and to treatment-related toxicity in patients with chronic Chagas disease [10–12]. Some of this evidence shows a central role of the host’s genetic background in regulating the immune response against the parasite, which leads to tissue damage when it is improperly regulated [13]. We hypothesized that this genetic background could make disease progression and toxicity outcomes more similar among siblings than among non-related patients and conducted a retrospective review to compare these variables between both groups.

## Results

From the 833 patients with a diagnosis of Chagas disease in our database, we identified 153 siblings in 71 groups. With the remainder of the database, 299 groups of non-siblings were randomly generated. The demographic and clinical characteristics of the full database and the sibling groups are shown in Table 1. Ninety-five percent of the patients were born in Bolivia. There was one case in which one brother was born in Bolivia and the other in Argentina. In another group, both brother and sister were born in Spain. Sixty-eight percent of the patients were female. Pre-treatment PCR assay was performed in a similar proportion in all the groups, but it was positive in a higher proportion among the sibling population than in the general and control population (40 *vs* 29%, *Z* = 2.64 and *Z* = 2.65, respectively; both *P* = 0.008). The proportion of patients to whom an esophagogram was performed was different (71% in the sibling group *vs* 61% in the control (*Z* = 2.27, *P* = 0.02) and 63% (*Z* = 1.81, *P* = 0.06) in the full database, although the rate of pathological esophagograms was similar in all groups (4–6%, without statistical significance).

**Table 1:** Demographic characteristics of the siblings grouped in 71 phratries, the remaining controls and the full cohort

Variable	Siblings (*n* = 53) (71 groups) (%)	Control (*n* = 685) (299 groups) (%)	*P-*value siblings *vs* controls	Cohort (*n* = 833) (%)	*P*-value siblings *vs* cohort
Country of origin					
Argentina	3 (1.96)	15 (2.18)	0.865	18 (2.16)	0.875
Bolivia	144 (94.11)	640 (93.43)	0.757	784 (94.11)	1.000
Brazil	0	2 (0.29)	0.505	2 (0.24)	0.544
Chile	0	1 (0.14)	0.141	1 (0.12)	0.668
Colombia	0	2 (0.29)	0.505	2 (0.24)	0.544
Ecuador	1 (0.65)	4 (0.58)	0.919	5 (0.60)	0.942
El Salvador	0	2 (0.29)	0.505	2 (0.24)	0.544
Honduras	3 (1.96)	5 (0.72)	0.152	8 (0.90)	0.240
Paraguay	0	6 (0.87)	0.247	6 (0.70)	0.299
Spain	2 (1.30)	1 (0.14)	0.028*	3 (0.36)	0.132
Venezuela	0	2 (0.29)	0.505	2 (0.24)	0.544
Sex					
Female	104 (67.97)	457 (66.71)	0.765	561 (67.34)	0.878
Male	49 (32.02)	223 (32.55)	0.899	272 (32.65)	0.878
Mean age (years)	42	44		44	
Pre-treatment PCR	112 (73.20)	495 (73.26)	0.988	607 (72.86)	0.931
Positive	45 (40.17)	200 (29.19)	0.008*	245 (29.41)	0.008*
Kushnir	142 (92.81)	637 (93.00)	0.934	779 (93.51)	0.749
K 0	119/142 (83.80)	539/637 (84.61)	0.803	658/779 (84.44)	0.841
K 1	18/142 (12.67)	77/637 (12.08)	0.840	95/779 (12.19)	0.868
K 2	2/142 (1.40)	12/637 (1.88)	0.686	14/779 (1.79)	0.734
K 3	3/142 (2.11)	9/637 (1.41)	0.525	12/779 (1.54)	0.609
Barium enema	128 (83.66)	553 (80.72)	0.399	681 (81.75)	0.572
Pathologic	24 (18.75)	119 (17.37)	0.685	143 (21.00)	0.527
Esophagogram	109 (71.24)	421 (61.45)	0.023*	530 (63.62)	0.069
Pathologic	6/109 (5.55)	26/421 (3.79)	0.322	32/530 (6.03)	0.818
Treatment	100 (65.35)	441 (64.37)	0.819	541 (64.94)	0.922
Benznidazole	91/100 (91)	409/441 (93)	0.392	500/541 (92.00)	0.678
Posaconazole	9/100 (9)	32/441 (7.2)	0.445	41/541 (7.57)	0.544
Rash (benznidazole)	16/91 (17.58)	88/409 (21.51)	0.279	104/500 (20.80)	0.3628

*P < 0.05 (statistically significant differences)

*Abbreviations*: *n*, number of patients; PCR, polymerase chain reaction

The results of the concordance analysis of the valid data are summarized in Table 2. Incomplete data from one or more of the members of a given group led to the loss of a notable number of groups in the analysis of each variable. We found a significant difference in the proportion of concordant results of the pre-treatment PCR assay (69% of the sibling groups *vs* 50% of the controls; *χ*^2^ = 4.73, *df* = 1, *P* = 0.02), and this difference was still significant after splitting the concordant groups into “all positive” and “all negative” of both siblings and controls, due to the difference in the “all positive”. Results of barium enema were more concordant among the siblings than among the controls, but the difference did not reach statistical significance (75 *vs* 62%, *χ*^2^ = 2.90, *df* = 1, *P* = 0.08), with similar rates of available data in both groups (67.60 *vs* 73.91%, *χ*^2^ = 1.15, *df* = 1, *P* = 0.28). The difference in concordance rates of DTH events related to benznidazole was very small between siblings and controls (62.5 *vs* 60.15%, *χ*^2^ = 0.06, *df* = 1, *P* = 0.80). In the analysis of the positive *vs* negative concordance in DTH, a substantial difference was found both in siblings and controls with more “all negative” groups among siblings than among controls (15.62 *vs* 3.91%, *P* = 0.04, OR = 3.95, 95% CI: 0.85–18.33), but none of these comparisons reached statistical significance. Cardiovascular evaluation was similar in both groups, mostly with negative results, resulting in high concordance rates (76.67% in the siblings and 73.4% in the controls, *χ*^2^ = 0.27, *df* = 1, *P* = 0.59). When considering esophagogram results the rates of concordant results were similar (89.47 *vs* 92%, *P* = 0.74, OR = 0.73, 95% CI: 0.19–3.41), despite a difference in the available data from relatives and non-relatives (53.52 *vs* 42.14%, respectively, *χ*^2^ = 3.01, *df* = 1, *P* = 0.08).

**Table 2:** Results from the two study groups

Variable	Siblings groups (*n* = 71)				Control groups (*n* = 299)				*P-*value	
	Available (%)	Concordant (%)	Positive (%)	Negative (%)	Available (%)	Concordant (%)	Positive (%)	Negative (%)	Conc	Pos/Neg
Pre-treatment PCR	39 (54.92)	27 (69.23)	12 (30.77)	15 (38.46)	172 (57.52)	86 (50.00)	23 (13.37)	63 (36.63)	0.023	0.015
Kushnir (>1)	60 (84.50)	46 (76.67)	3 (5.00)	43 (71.67)	297 (99.33)	218 (73.4)	6 (2.02)	212 (71.38)	0.599	0.331
Esophagogram	38 (53.52)	34 (89.47)	–	34 (89.00)	126 (42.14)	116 (92.06)	–	116 (92.06)	0.740	0.740
Barium enema	48 (67.60)	36 (75.00)	3 (6.25)	33 (68.75)	221 (73.91)	137 (61.99)	8 (3.62)	129 (58.37)	0.088	0.160
DTH	32 (45.07)	20 (62.50)	15 (46.88)	5 (15.62)	128 (42.80)	77 (60.15)	72 (56.25)	5 (3.91)	0.808	0.064

*Notes*: The first column of each group reflects the availability of data, meaning that data were complete for the two or three members of each phratry or randomly-generated control group. When data of one or more members of the same group were missing, that group was discarded for statistical analysis. The *P-*values are recorded in the last two columns. The first column (Conc) is the result of comparing concordance among siblings against that among control groups. The second column (Pos/Neg) is the result of comparing the same results differentiating those with concordant positive or negative results

*Abbreviations*: n, number of groups

## Discussion

In our retrospective study we found a statistical difference in the rate of concordance of pre-treatment PCR, but not in the remaining variables (Kuschnir status, result of the esophagogram and barium enema, and the presence of DTH after benznidazole use).

In the studied data, there was a near 2:1 ratio of female to male patients, which may reflect patterns of migration and approach to healthcare, probably under-representing male involvement in the endemic countries. The vast majority of patients were Bolivian, so the results are applicable mainly to this population. There was a significant difference in the country of origin only in the case of Spanish patients, which represented documented cases of vertical transmission. They were so small in number that the likely effect on the global analysis is negligible (2 in the sibling groups and 1 in the controls, *P* = 0.02).

### PCR

The main and only significant difference was found in the concordance of pre-treatment PCR. Nevertheless, the significantly higher positive result in pre-treatment blood PCR test among the patients included in the sibling groups as compared to the controls (40% *vs* 29%, *P* = 0.008) may have biased the results; it must be noted that when splitting the concordant groups on the basis of positive or negative results, the biggest difference was seen between the “all positive” sibling and control groups, with a narrower difference between the “all negatives”.

Taking into account these limitations, the difference in the concordance of PCR results between siblings points to the presence of genetically determined mechanisms involved in the relationship between the host and infecting *T. cruzi*. There is growing evidence for the influence of genetic elements on parasite control by the immune system in patients with chronic Chagas disease, but these studies are focused on end-organ damage rather than on the detection of the relationship of PCR assays and persistent parasitaemia because of the limitations of the technique. It is known that parasitaemia oscillates during the natural history of the disease, and a positive blood PCR result only indicates that in the moment of the sampling, there was parasitic DNA in the bloodstream. Furthermore, some acquired conditions are known to modify the results of PCR assays in patients with Chagas disease, such as co-infection with helminths, infection with human immunodeficiency virus, immunosuppression and pregnancy [14–17]. In the last 15 years a strong effort has been made in validation and standardization of these techniques, reaching a sensitivity of 60–90% for the best performing methods, with a specificity of 100% [18, 19]. In our study, pre-treatment PCR was performed in 73% of our patients. It was positive in 29.41% of the patients, the expected sensibility using available assays in a clinical setting. The difference between the best performing methods and the results obtained in our cohort can be explained by the different sample volume, conditions of conservation of the sample, the methods used to isolate DNA, the sequences and the reagents used, and the thermo-cycling conditions [18].

### Cardiomyopathy

We did not find a significant relationship between kinship and the presence of chagasic cardiomyopathy. The immune response regulation against the parasites infesting the myocardium seems to be of central importance in the development of tissue damage. In patients with cardiomyopathy, the upregulation of this response leads to the deployment of a high-grade, harmful inflammation [20, 21]. Familial aggregation of chagasic cardiomyopathy in a cohort of patients with serologically confirmed Chagas disease including 247 patients with cardiomyopathy and 345 controls suggests a genetic component in the regulation of this immune response [22]. Different HLA class I and II polymorphisms have been identified as protective or predisposing factors to the development of chagasic cardiomyopathy in different Latin-American populations (i.e. a higher frequency of chagasic cardiomyopathy with DRB1∗01, DRB1∗08, and DQB1∗0501 in Venezuelan patients, and with HLA-DQ1 in Brazilian patients). Mutations of the killer cell immunoglobulin-like receptor genes, TNF-α and caspase pathways have also been related to cardiac involvement [23–27].

As cardiovascular disease (sudden death, end-stage cardiac failure and thromboembolism) is the leading cause of death in patients with Chagas disease, a cardiac screening should be performed in all patients [11, 28]. Despite that aim, in our cohort the missing evaluations invalidated 15.5% of the sibling groups (only 0.67% of the control groups). It is also relevant that cardiac abnormalities have a low prevalence in our cohort (121 patients, 15.56%, are rated in a Kushnir stage other than 0), and notably most concordant groups in both siblings and controls are due to “all negative” concordance thus making unapparent the familiar aggregation of cardiomyopathy reported in the aforementioned studies.

### Gastrointestinal involvement

When considering barium enema, concordance was higher among the siblings than among the controls, but the difference did not reach statistical significance (75 *vs* 62%, *P* = 0.08). As in the case of the cardiac evaluation, there are few “all positive” groups. Regarding the esophagogram, the rates of concordant results were similar (89.47 *vs* 92.00%), despite a difference in the available data from relatives and non-relatives (71 *vs* 61%). These findings may be explained because most studies were normal in both groups (indeed, there are no “all positive” groups).

Although small, the difference in the results of the concordance of the colonic and the cardiac involvement in our study may reflect different tissue tropism of *T. cruzi* discrete typing units (DTU) and/or different damage pathways for the digestive and the cardiologic form. On one hand, megaesophagus and megacolon are seen mainly in patients from the south cone of America and they have been related to specific pathogenicity of the DTU present in this area [29]. On the other hand, the pattern of the inflammatory infiltrates in the gastrointestinal tissue differ from those described in the cardiac form of chronic Chagas disease (inverse CD4/CD8 ratio, diminution of CD3+ and CD19+ cells) [30]. Indeed, the non-classical major histocompatibility class I chain-related genes A and B (*MICA* and *MICB*) seem to rule the interaction of Tγδ, Tαβ CD8+ and natural killer cells with *T. cruzi* infesting the neuronal and muscular layers of hollow viscera [31]. The association of MICA*011 and HLA-B*14 and DRB1*01 could relate to megacolon [32].

The low rates of end-organ damage detected among the patients in our cohort may mask many differences between siblings and controls. These low rates may be explained because the vast majority of our diagnoses are screening tests of healthy individuals with a mean age of 44 years old, and with female predominance, so they lack the classical risk factors for the development of the determinate phase of chronic Chagas disease [33].

### DTH

Our study does not show a higher concordance in DTH among siblings. Within those treated with benznidazole, DTH was registered in 17% of the relatives and in the 20% of the non-relatives controls. In previous prospective studies and clinical trials DTH appeared in a 25–50% of the patients [6–9, 34]. This difference may be explained by the incomplete report of mild to moderate adverse events, and the large number of patients lost in the follow up or who have not completed treatment because of adverse events different from skin reactions, or reasons not related to toxicity in our retrospective cohort. Indeed, the incidence found in this study is similar to that reported of serious adverse events leading to treatment interruption, which are more likely to prompt medical assistance and therefore to be registered in clinical records.

Cutaneous DTH has been related to a delayed hypersensitivity with a Th2 profile (with higher levels of IL-5, IL-10 and eosinophils). A previous study suggested an association between the presence of HLA-B*3505 and moderate-to-severe DTH. This variant is relatively frequent among the Bolivian population [10]. Interestingly, although not significant, when splitting concordant groups into “all positives” and “all negatives”, a bigger proportion of all negatives was found among sibling groups (15 *vs* 4%). This finding may reflect a lower prevalence of HLA-B*3505 and other genetically determined toxicity risk factors.

It must be noted that the results of the aforementioned studies about the genetic determinants of end-organ damage in chronic Chagas disease have highly heterogeneous results, even between cohorts of patients from the same country. This is probably due to small sample sizes, lack of standardization in study methods (laboratory and statistical), ethnical variations and parasite variability [25, 35–41]. The overall immune response against persistent *T. cruzi* infection is likely related to polygenic mechanisms, as it depends on a complex cell and cytokine crosstalk [13, 24, 42–44], making it difficult to obtain valuable conclusions from the analysis of the relationship between a single genetic locus and a disease phenotype.

Certain limitations must be considered to contextualize the results of our study. First, as 94.11% of our patients came from Bolivia, our cohort had a highly homogeneous population, which may have masked the influence of familiar genetic background on clinical outcomes between siblings. Thus, our results may not be applicable to patients from underrepresented geographical origins. Similarly, as the origin of our patients was geographically narrow, they may have been infected with DTU with similar tissue tropism. Unfortunately, information about strain identification was not available in our cohort as it is not a part of our usual practice. Secondly, ambiental factors and re-infections are clear modifiers of the natural history of chronic Chagas disease [45]. In our cohort, re-infections had been interrupted due to an improvement in vector control strategies in the countries of origin and to migration to an area where vector-borne transmission had not yet been reported. This could have induced a slower progression of organ damage, thus masking different outcomes in this population.

## Conclusions

We found a significantly higher proportion of concordance in the result of pre-treatment PCR among Bolivian siblings. A non-significant trend was noted in barium enema results and the absence of DTH related to benznidazole. Prospective studies with bigger sample sizes may be necessary to identify significant differences in end-organ involvement of chronic Chagas disease.

## Methods

### Study design and availability of data

We performed a single-center retrospective study based on the database of patients who received the diagnosis of Chagas disease between 2002 and 2015 at the Vall dʼHebron University Hospital, a tertiary care center included in the International Health Program of Catalonian Health Institute (Programa de Salut Internacional de l’Insitutt Catalá de Salut, PROSICS), in Barcelona, Spain (see Additional file 1: Table S1).

Data of 833 patients on the results of blood PCR for *T. cruzi* before treatment inception, the presence of organic damage (cardiac and/or gastrointestinal), and the presence of DTH in patients treated with benznidazole were reviewed. We identified groups of siblings and used the remaining patients of the database as controls, randomly generating non-related patient groups of 2 or 3 individuals in a ratio of 4:1 compared to groups of siblings. After the composition of these groups, we anonymized the data prior to analysis. We analyzed whether the variants were concordant or not in sibling groups and in the randomly-generated non-sibling groups.

### Clinical assessment

Chagas disease was diagnosed by means of two serologic tests: one based on recombinant antigen (Bioelisa Chagas, Biokit, Lliçà d’Amunt, Spain) and the other based on crude antigen (Ortho *T. cruzi* ELISA, Johnson & Johnson, High Wycombe, UK) [46]. A polymerase chain-reaction (PCR) to detect *T cruzi* DNA in peripheral blood was performed in most patients according to the method described by Piron et al. [47].

At the time of the diagnosis, patients were evaluated with a physical examination, an electrocardiogram (ECG), a chest x-ray and, if there was an abnormal result, cardiac ultrasound. Patients were classified according to the Kuschnir classification [48]. Gastrointestinal assessment was based in symptoms present at initial evaluation and barium radiographic imaging of esophagus and colon according to Rezende and Ximene’s classifications. Patients younger than 55 years were offered treatment with benznidazole. It must be noted that some patients were treated with posaconazole during a previous study [49]. Medication-related adverse events were registered prospectively. For statistical analysis, only cutaneous toxicity was considered as there is some evidence about genetic factors influencing this adverse event.

### Statistical analysis

In each sibling and non-related randomly generated group we evaluated the result of the PCR (positive or negative), Kushnir (grade 0 *vs* 1 or higher), esophagogram (pathologic or normal), barium enema (pathologic or normal) and the presence of DTH within its members. The possible results were concordant, discordant or missing when data of one or more of the members of the group were not available. Those groups in which data of the considered variable were missing in one or more of its members were excluded for statistical analysis. Then we compared the difference in the proportions of concordance between the siblings and the control group using Fisher’s exact test and Pearson’s Chi-square test. We repeated the test splitting the concordant groups into “all positive” and “all negative”. All data were analyzed with Stata v.13.1 software and R v.3.5.1 software.

## Additional file

**Additional file 1: Table S1.** The deidentified full dataset used in the study. Last column “Phratry” indicates with a 0 those patients who had no relatives within the database and identifies with a number the different sets of siblings.

## Data Availability

The dataset supporting the conclusions of this article is included within the article and its additional file.

## Abbreviations

DNA: deoxyribonucleic acid
DTH: delayed type hypersensitivity
DTU: discrete typing units
HLA: human leukocyte antigen
OR: odds ratio
PCR: polymerase chain reaction
PROSICS: programa de salut internacional de l’Institut Catalá de Salut (International Health programme from the Catalan Health Institute)
TNF: tumor necrosis factor
